# New Methods of Administration of Ether

**Published:** 1909-02-06

**Authors:** 


### Anæsthetics.

### NEW METHODS OF ADMINISTRATION OF ETHER.

With the single exception of nitrous oxide gas, there can be little doubt that the safest general anæsthetic yet discovered is ether. All authorities agree upon this point, however much difference of opinion there may be between them on the relative merits of other anæsthetic drugs. It does not, of course, follow that ether is the best anæsthetic to use for every major operation. The considerations which guide the choice of an anæsthetic in various kinds of operations have already been discussed in these columns; it is therefore unnecessary to review them, but before setting out some recent innovations in the technique of ether administration, it will be well to mention one or two of the drawbacks of ether as usually exhibited.

The well-known Clover's inhaler is the apparatus by which, in this country at least, ether is administered ninety-nine times out of a hundred. Its merits are familiar to all from student days onwards. It allows of a highly concentrated vapour being inhaled, especially when re-breathing is permitted, and this concentration can be varied at will by a simple mechanism; it is admirably adapted for the gas-ether and ethyl-chloride-ether sequences, and it stands hard wear well. But it has certain defects. An unnecessary and injurious degree of cyanosis is very easily produced. In the hands of careless or inexperienced anæsthetists this occurs much too often, and though it is very seldom the cause of an actual fatality on the table, such an event does happen, perhaps less infrequently than is usually supposed. It is an apparatus impossible to keep even moderately sterile. The bag, the face piece, and the inhaler itself must inevitably swarm with pathogenic bacteria of all kinds after very short use. How far Clover's inhaler is responsible for " ether pneumonia " there are no data for ascertaining, but at least it is a septic piece of furniture for a modern operating room. It has other disadvantages, such as a terrifying appearance, which we need not now deal with.

Various substitutes have been suggested for this inhaler. Those known by the names of Ormsby and Rendle have had most vogue, but neither has supplanted Clover's inhaler for routine use. Extreme concentration of the vapour by some method involving, however slightly, limitation of air entry, has been a common feature of these devices. To induce anæsthesia by giving ether on a lint mask, such as is in general use for chloroform and chloroform mixtures, was supposed to be impossible for all but exhausted and enfeebled patients. Thus Hewitt, when mentioning the " open " method in some of his earlier writings, says: " It is wasteful and inferior to others in general use. It may be advantageously chosen, however, in cases in which air limitation would be open to objection, and in those in which very little anæsthetic is needed." Only a year ago it was stated in a new text-book that to " attempt to anæsthetise a robust patient by means of ether poured on lint would result in the formation of much snow on the lint, the using up of several half-pint bottles of ether, and would terminate in failure." It has also been held that there is more risk of producing catarrhal and pulmonary affections with semi-open methods than with Clover's inhaler.

In America, however, there are many enthusiastic advocates of the open-ether method. They do not allow that the pessimistic forecast of the last quotation is justified by actual experience, and they claim that by giving ether on a gauze or lint mask they can obviate several of the gravest objections to which the closed methods are open. In its simplest form the procedure is as follows. Upon an ordinary Skinner's mask are stretched eight or ten thicknesses of plain sterilised unmedicated gauze. Under the edges of the mask, wherever it does not fit the face exactly, gauze is gently packed, so that the patient shall not through these apertures breathe air uncharged with ether vapour; a little vaseline is useful where the mask rests upon the skin. The patient is prepared in the ordinary way and is supine; all the usual precautions to secure a proper airway must be taken. Ether is then dropped upon the gauze, at first slowly, and then more and more liberally as the patient becomes accustomed to the novelty of inhaling its pungent vapour. If he can be persuaded to breathe deeply and regularly, a healthy adult can thus be anæsthetised to the full surgical degree in from ten to twenty minutes by the use of about two and a half to five ounces of ether. Women, and especially those of either sex who are not in robust health, are more easily anæsthetised.

To keep a patient thus anæsthetised for an abdominal section, let us say, will require the use of from six to twelve ounces of ether per hour, for the liquid evaporates rapidly from the mask, which must be kept almost constantly wet with the drug. It is evident, therefore, that the method is more wasteful and expensive than that of Clover. But the advantages claimed for it are very considerable. It is evidently a much cleaner process; the gauze is thrown away after use, and the mask can be dropped into a sterilser. Cyanosis is only possible if the patient's airway becomes obstructed; the apparatus cannot contribute to any such result. Bronchial and pulmonary complications are said to be much less frequent than by any other method of administering ether. A fatal result from overdose is said to be impossible. Vomiting is rarely troublesome. How far these claims can be substantiated it is too early yet to decide, but if they are in substance and in fact true, a great advance has undoubtedly been made.

As with other anæsthetics, it is during the induction period that difficulties are especially prone to arise. A stage of excitement is common, during which unconscious acts of violence may require restraint. There is at least no danger in pushing the anæsthetic vigorously when this happens, as there unquestionably is at a similar stage of chloroform induction. With the robust and the alcoholic the induction takes a long time, which delay may be obnoxious to the operator. Indeed, in rare cases it is impossible to produce anæsthesia at all by the unmodified open method; but this objection can be got over in a way to be presently described. To many patients the smell of ether is repugnant, either actually or by its associations; and if they open their eyes the vapour causes smarting. As a matter of fact, patients almost always shut their eyes of their own accord, and the irritating effects of ether vapour on the nose and throat are a good deal exaggerated. Personally, the writer is gratified rather than offended by inhaling ether thus dropped on a gauze mask.

When once anæsthesia has been brought about, it is easily maintained; relaxation can be obtained sufficient for the performance of any operation. The secretion of mucus is in most cases not very copious, certainly not so great as with Clover's apparatus. Vomiting is no more troublesome, indeed probably much less so, than after chloroform. The regurgitation of mucus immediately after the suspension of the anæsthetic is common, but even that is often missed, and serious post-anæsthetic vomiting is little to be feared.

There would seem to be fewer contra-indications to ether by the open method than to the same drug in closed machines. Operations on the face or mouth are evidently out of the question; but whether for empyema, trephining, and thyroidectomy, for instance, the superiority of chloroform can be maintained, as it can be against the closed methods, remains to be seen. For abdominal sections open ether is excellent when once the prolonged induction has been successful. In fact, when an exhausting laparotomy is being undertaken in a patient of poor vitality under chloroform or C.E. mixture, it is frequently of the utmost advantage, should the pulse flag, to change to ether on the mask.

To get over the disadvantage of the long-induction period, with the possibility of much struggling or of failure to induce anæsthesia, some of the American anæsthetists are now recommending the ethyl-chloride-ether sequence, given on the gauze mask. They spray ethyl chloride on to the gauze, holding the mask at first a little distance from the face, and then drop ether on also, placing the mask right down on to the face. The ether is not to be substituted abruptly for ethyl chloride, but is gradually intermingled with it in increasing proportion. The total amount of ethyl chloride used is about ten c.c., which is more than can be safely given in the closed apparatus in common use in Britain for this drug. Moreover, the quantity of ether necessary to produce surgical anæsthesia is a good deal diminished, and time is, of course, also saved. In experimenting with this sequence the writer has found that in some cases everything goes like clockwork, but that in others there is struggling and delay just as with open-ether pure and simple. Probably in these cases more ethyl chloride should be used. Fifteen c.c. does not seem to produce any bad effects when thus given with free air supply and with commixed ether.

The preliminary use of morphine, or of morphine with atropine, has also found favour with some administrators when employing the open-ether method. A quarter of a grain of morphine is given half an hour before the time fixed for the operation. Less anæsthetic is required to produce anæsthesia, and, generally speaking, a less profound degree of anæsthesia will suffice for the purposes of the surgeon. Post-anæsthetic vomiting is also said to occur less frequently. A disadvantage is that some patients, when thus semi-narcotised with morphine, do not breathe deeply enough during induction to inhale the requisite quantity of the drug. The effect on the pupils of these two alkaloids must also be remembered and allowed for.

The latest new device in the way of new modifications in ether administration hails from Berlin, and is the invention of Dr. Klapp. This observer has shown by experimental work upon animals that when the limbs are constricted with elastic bandages in such a way as to stop the circulation in them, and so to reduce the quantity of blood circulating through the cardio-pulmonary circuit, a much smaller amount of anæsthetic will suffice to produce unconsciousness. In addition to this, when the administration is suspended and the bandages loosed, the restoration of the blood in the limbs which is uncharged with anæsthetic causes a rapid recovery of consciousness. These results have been applied to human beings by Dr. Zur Verth, who reports very favourably on the method. The legs only were bandaged, as it was feared that the nerves of the arms might suffer from compression against the bones if the upper extremity were utilised. It is said that strong men when thus treated are easily anæsthetised by the open-ether method, that recovery is rapid when the bandages are released, and that no bad effects are observed in the limbs.

It is suggested also that the advantages are not confined to ether administrations, but apply just as well when chloroform or mixtures are being used. Indeed, the conjecture is put forward that a rapid and immediate auto-transfusion can be obtained by the simple process of cutting off the bandages whenever any difficulty or danger seems to require any such measure. The hypothesis is that to add to the circulation a quantity of blood rich in carbonic acid and poor in chloroform is to provide a powerful stimulus to the respiratory centre. If this plan is found to bear out what is claimed for it, it seems certainly preferable to any form of morphine narcosis. Those interested in the subject will find Dr. Zur Verth's paper in the *Münchener Medizinische Wochenschrift* for November 15, 1908.

Acidosis following chloroform anæsthesia has been for some time familiar as a subject of discussion among anæsthetists. It appears from the researches of Messrs. Wallace and Gillespie* that this condition is also very frequent with ether, and much more so after closed than after open administration. They conclude that secondary vomiting after anæsthesia is in direct relation to the quantity of acetone produced: that all anæsthetics should be given by open methods: that in prophylaxis glucose is more valuable than sodium bicarbonate: and that in all cases of vomiting lasting over twelve hours the stomach should be washed out with a solution of sodium bicarbonate, some of which should be left in that organ. Calomel in repeated small doses is also advised.

* *Lancet*, December 5, 1908.

